# Natural and Synthetic Polymers for Bone Scaffolds Optimization

**DOI:** 10.3390/polym12040905

**Published:** 2020-04-14

**Authors:** Francesca Donnaloja, Emanuela Jacchetti, Monica Soncini, Manuela T. Raimondi

**Affiliations:** 1 Department of Chemistry, Materials and Chemical Engineering “Giulio Natta”, Politecnico di Milano, 20133 Milan, Italy; Emanuela.jacchetti@polimi.it (E.J.); Manuela.raimondi@polimi.it (M.T.R.); 2 Department of Electronics, Information and Bioengineering, Politecnico di Milano, 20133 Milan, Italy; Monica.soncini@polimi.it

**Keywords:** polymeric scaffold, natural polymer, synthetic polymer, bone tissue engineering, bone tissue regeneration

## Abstract

Bone tissue is the structural component of the body, which allows locomotion, protects vital internal organs, and provides the maintenance of mineral homeostasis. Several bone-related pathologies generate critical-size bone defects that our organism is not able to heal spontaneously and require a therapeutic action. Conventional therapies span from pharmacological to interventional methodologies, all of them characterized by several drawbacks. To circumvent these effects, tissue engineering and regenerative medicine are innovative and promising approaches that exploit the capability of bone progenitors, especially mesenchymal stem cells, to differentiate into functional bone cells. So far, several materials have been tested in order to guarantee the specific requirements for bone tissue regeneration, ranging from the material biocompatibility to the ideal 3D bone-like architectural structure. In this review, we analyse the state-of-the-art of the most widespread polymeric scaffold materials and their application in in vitro and in vivo models, in order to evaluate their usability in the field of bone tissue engineering. Here, we will present several adopted strategies in scaffold production, from the different combination of materials, to chemical factor inclusion, embedding of cells, and manufacturing technology improvement.

## 1. Introduction

The elderly population has been increasing throughout the years with a consequent increment in bone-related diseases. Bone tissue fractures or loss cause mobility limitation and severe disabilities that represent one of the major public health problems. In this social context, one of the most promising strategies for bone injury treatment is regenerative medicine, and nowadays, research has been focusing on scaffold optimization in order to guarantee extra cellular matrix-like support to the cells [1]. The relevant role of the emerging scaffold-based strategy in the bone tissue regeneration field will be highlighted after a brief introduction concerning the bone’s structure and composition, its physiological remodeling, and conventional therapies. We propose a review of the most promising polymeric material with particular attention paid to the widespread adopted strategies to design scaffolds suitable for bone tissue regeneration.

### 1.1. Anatomy

Bone is a dense connective tissue, which serves a variety of functions. It provides support and protection of soft tissues and represents the mineral reserve of the body [2]. From a microscopic point of view, it is possible to distinguish primary (or immature) bone from the secondary (or mature) one. Primary bone is the first bone tissue to appear in embryonic development and in repair processes such as fractures. It is characterized by random disposition of fine collagen fibres, in contrast to the well-organized lamellar disposition of collagen in the secondary bone. Primary bone tissue is usually temporary and it is replaced by secondary bone tissue in adults, with the exception of a very few places in the body (e.g., near the sutures of the flat bones of the skull, in tooth sockets, and in the insertions of some tendons). In addition, primary bone tissue presents lower mineral content and a higher proportion of osteocytes than the secondary bone tissue. The secondary bone is on turn constituted of the cortical bone, that comprises 80% of the skeleton and it is characterized by low porosity (5–30%) and the trabecular one, with high porosity spanning from 30% to 95%. Native bone tissue physical characteristics are summarized in Table 1 [3,4]. The functional unit of the mature bone is the osteon, also called the Haversian system. It is characterized by a cylindrical shape and collects the blood vessels in its central canal: the Haversian canal. The walls of Haversian systems are formed of concentric lamellae. Lamellae are organized in parallel aggregates or distribute randomly in cortical and trabecular bone, respectively. Lamellae constitute of cells and intercellular substance. The latter is composed of mineralized layers, interposed to the organic ones, that contribute in cushioning solicitations. The inorganic fraction constitutes the 77% of the total matrix and consists of calcium phosphate (90%) and calcium carbonate (10%). The organic fraction (23%), also called “osteoid”, is instead mainly composed of collagen type I (the most widespread in the human body among the approximately 29 existing types) fibers, displaced in an amorphous matrix (90%), and some other proteins. By volume, bone consists of 36% inorganic component, 36% organic component, and 28% water. While organic fibers confer toughness, the inorganic fraction guarantees hardness to the bone. Instead, the water content confers viscoelasticity property to the bone [5,6].

### 1.2. Cellular Components

Bone exhibits four different cell types: osteoblasts, osteoclasts, bone lining cells, and osteocytes.

The first three cell types derive from local mesenchymal progenitor cells and are located along the bone surface. Instead, osteocytes permeate the interior of the bone and derive from the fusion of mononuclear blood-bone precursor cells [6].

During bone tissue formation, mesenchymal cells differentiate in osteoprogenitor cells that, after proliferation, differentiate into the osteoblasts. These latter cells deposit organic components of the bone matrix and, finally, became osteocytes [5]. Osteocytes represent the most abundant cells of the bones (90–95%) and are characterized by a lifespan of up to 25 years. They are located within lacunae, entrapped within the calcified matrix, and their shape is tissue-specific dependent: osteocytes from cortical bone display an elongated morphology in comparison to the ones from trabecular bone [2]. Due to their ability in sensing mechanical pressure and load, the osteocytes act as mechanosensors regulating the osteoblasts and osteoclasts activities in bone remodeling [2]. The osteoclasts are polynucleate giant cells deriving from haemopoietic stem cells, and their precursors are part of monocyte macrophagic lineage. Osteoclasts function is to digest the bone matrix [5]. This process happens in three main phases: the adhesion of osteoclast to the matrix, acid dissolution of the mineral matrix, and enzymatic digestion of the organic matrix. Finally, osteoblasts are quiescent flat-shaped bone lining cells that cover the bone surfaces. Even if their functions are not completely elucidated yet, it is known that they are involved in osteoclast differentiation and act to prevent the direct interaction between osteoclasts and bone matrix [2].

### 1.3. Bone Mechanical Properties

In general, bone tissue behaves as an anistopic material, characterized by elastic modulus of 18 GPa in case of axial force application, 12 GPa in trasverse load condition and, finally, reacts with only 3.3 GPa to shear stress [7]. In addition, bone mechanical response changes also according to the versus of the sollecitation, showing a compressive strenght that ranges from 12.56 to 16.89 kg/mm^2^, and on a tensile strenght of 10–12 kg/mm^2^ [7].

The specific bone mechanical properties are mainly affected by both the inorganic/organic matrix ratio, responsible for hardness and elaticity, and the cortical/trabecular one [7]. Indeed, despite the similarity in terms of materials and morphological features between the cortical and trabecular bone, the differences in porosity confer them peculiar mechanical properties. In particular, while the cortical bone is characterized by high compressive strength (100–230 MPa), the trabecular bone stregth is one or two order of magnitude less (2 to 12 MPa) [3,7]. On the other hand, the trabecular bone reacts with high energy storage capability that reflects on increases of its length up to an order of magnitude higher than the cortical one (50% vs 2%) [7]. Table 1 summarizes the two the two bone types differences, relevant for bone tissue engineering application.

### 1.4. Homeostasis

Bone is continuously renovated and remodeled during the whole life, although its rate of change is considerably slower in adults. These processes are subjected to both mechanically- and metabolic-regulated homeostasis, which regulates the calcium concentration in the plasma and guarantees the mechanical functions. When an external force causes a strain state higher than 2500 μstrain (0.25% deformation), osteoblasts act deposing new bone; on the other hand, in case of too low sensed strain (<50 μstrain) osteoclasts reabsorb the bone matrix. In addition, bones are used as calcium reserve to maintain the extracellular fluid calcium level within the physiological range (8.5–10.5 mg/100 mL of blood in adults). The parathyroid hormone and calcitonin are two hormones secreted by the parathyroid glands involved, together with bone, in the regulation of the extracellular calcium concentration. In fact, when the calcium concentration drops down in the plasma, the parathyroid hormone is released and acts both increasing osteoblast proliferation and differentiation and reducing osteoblast apoptosis, resulting in a new production of calcium [9]. Vice versa, when calcium concentration in the plasma increases, calcitonin inhibits the bone reabsorption, blocking the osteoclast activity. Lastly, when the necessity of calcium and phosphate decreases in the bone tissue, osteocytes release factors that interact with the kidneys stimulating the phosphaturia, which in turn inhibits calcium absorption at the intestinal level [5].

### 1.5. Spontaneous Repair

Bone tissue is characterized by osteogenic processes, which are effective at repairing and restoring bone tissue after injuries. Osteogenesis occurs after inflammatory process during which fibroblasts and macrophages form a granulation tissue around the lesion [10]. The granulation tissue is then colonized by mesenchymal cells from the bone marrow [5]. Immediately adjacent to the fracture line, a cartilaginous callus is formed. At the edges of the new cartilage tissues the periosteum swells and primary bone formation is initiated [10]. Gradually, the spongy immature bone is reabsorbed through an internal remodeling process and it is replaced with lamellar mature bone [5]. Despite its self-healing ability, in the presence of large (>5 cm) defects (i.e., non-union fractures, tumor ablations, maxillofacial trauma or degeneration), bones cannot self-repair and reconstructive techniques and cellular therapies are required [11,12].

### 1.6. Bone Tissue Pathologies and Conventional Therapies

In addition to the trauma, other diseases affect the bone tissue such as tumors, infections, osteopetrosis, pseudoarthrosis, osteoporosis and others [4,11,13,14,15]. Conventional therapies for osteoporosis and osteopetritis include both non-pharmacological and pharmacological approaches. Non-pharmacological guidelines include proper calcium and vitamin D intake, weight bearing exercise, smoking cessation, limitation of alcohol/caffeine consumption and fall-preventing techniques [16]. Instead, the pharmacological approach involves antiresorptive medications and anabolic medications aiming at simultaneously decreasing the bone reabsorption and promoting bone formation, respectively. Nevertheless, antiresorptive agents and hormones administration are cause of cardiovascular, intestinal, renal and urinary system side effects. In this context, long-term drug-delivery materials could represent an alternative strategy to guarantee local release of such medications. Moreover, in case of fractures, major size bone defects or pathologies requiring bone surgical resection (i.e., osteosarcoma, chondrosarcoma, Ewing sarcoma) are usually treated with external fixation, metallic prostheses or bone grafts [17,18]. Nevertheless, several drawbacks related to stress shielding situations, allergies, septic and aseptic mobilization, periprosthetic osteolysis, structural and fatigue subsidence make scaffold-based regenerative medicine one of the most promising strategy [19]. In this scenario, the continuous increment in bone-related diseases due to population ageing represents an additional stimulus in developing artificial scaffolds able to substitute the physiological ones (autograft, allograft), whose availability is limited.

## 2. Scaffold-Based Regenerative Medicine

**Figure 1:**
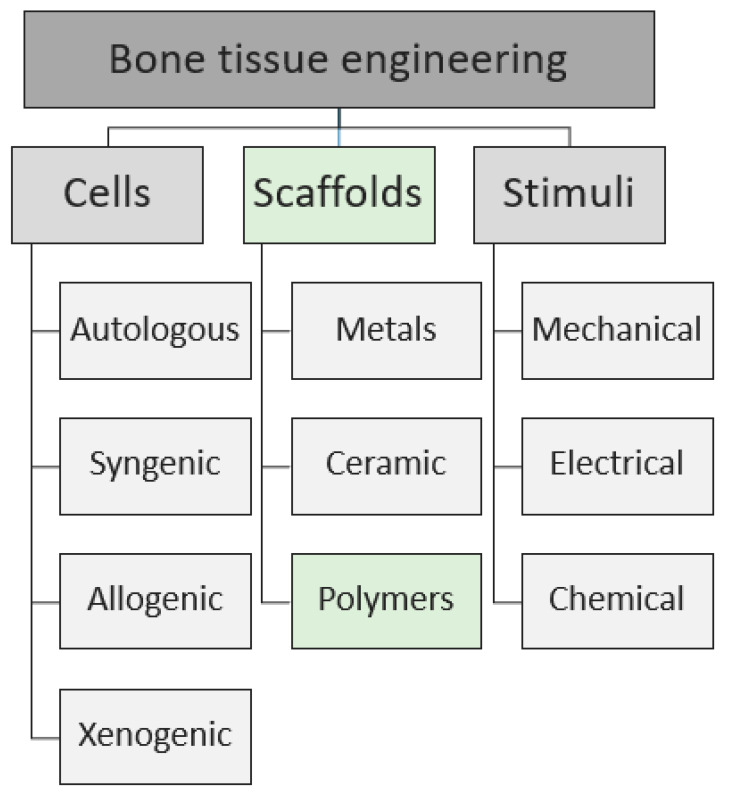
Schematic representation of the main components of the bone tissue engineering. In this review we will focus on the natural and synthetic polymeric scaffolds.

**Figure 2:**
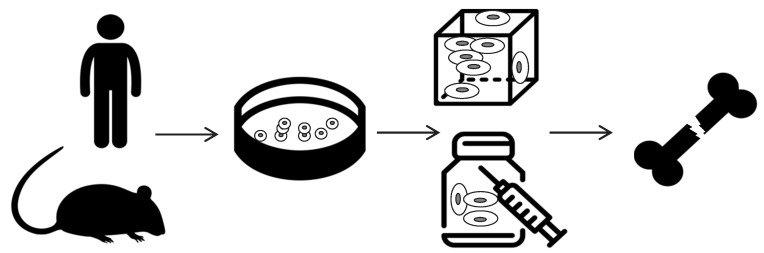
Schematic representation of the bone tissue engineering strategy. Cells are harvested from either human being or animals and then they are expanded in vitro. If required, cells are differentiated and then are seeded into a rigid or injectable scaffold. Finally, cellularized scaffolds or hydrogels are implanted or injected in patients.

Tissue engineering is an interdisciplinary field that applies the principles of engineering and life science toward the development of biological substitutes aiming at creating therapeutic “strategies” to restore, maintain, or improve the tissue functions [20]. Indeed, bone tissue engineering aims at repairing and promoting regeneration of new tissue, by the combination of three main components, schematized in Figure 1: scaffold, cells, and chemo-physical stimuli. The cells can be obtained from a wide variety of sources (autologous, syngenic, allogenic, and xenogenic), and several scaffolds made by different materials can be used in order to guarantee specific mechanical properties of the substrates. Basically, the scaffold-based strategy (illustrated in Figure 2) consists of:

1. cells harvesting from human being (named “autologous” source if patient and donor coincide or “allogenic” if they differ) or animals (termed “xenogenic” source), their in vitro expansion and, eventually, their differentiation;
2. cells seeding on optimized scaffold, designed and developed to reproduce the in vivo bone feature;
3. cellularized scaffold implantation into the patient damage site.

Nowadays, the main limit in tissue engineering is the optimization of scaffolds able to mimic the cells physiological condition. In this review, after a short description of the cells and stimuli most used in bioengineering, we will focus on the natural and synthetic polymeric scaffold component.

### 2.1. Cells Source

Cells are usually classified based on their potency. They could be totipotent, pluripotent, multipotent, or unipotent cells, according to their ability to differentiate into any cell phenotype, in multiple cell families, into a closely related families, or into a single cell type, respectively. In the tissue engineering field, other criteria have to be considered in the cell choice, among which their availability, their proliferative capability, their source and related immunogenicity. In this context, even if the totipotent and pluripotent cell sources represent the most attractive strategies in terms of differentiation and proliferative capability, the embryonic or adult germ progenitor cells usage is limited by both ethical issues and the limitations of the differentiation protocols. On the other hand, while the use of adult autologous unipotent cells is limited by their reduced availability and expandability, the allogenic source would require strong immunosuppressive therapy with severe consequences for the patient. Considering these drawbacks, the most promising cells are the autologous mesenchymal stem cells (MSCs) [21]. They are very effective in bone regeneration and, being multipotent cells, they are able to differentiate in different phenotypes (i.e., chondrogenesis, myogenesis, tendogenesis, ligamentogenesis, marrow stromal, adipogenesis) [22]. In particular, in bone tissue engineering field, several cells have been exploited such as adipose-derived stem cells (ADSCs) or birth-associated perinatal tissue umbilical cord (UMSCs). Another big family of cells that can be used is the one of dental origins, such as dental pulp stem cells (DPSCs), periodontal ligament stem cells (PDLSCs), gingival MSCs (GMSCs), and dental follicle stem cells (DFSCs) [23]. Some studies are also employing jaw bone mesenchymal stem cells (JBMSCs) [24], amnion mesenchymal stem cells (AMSCs) [25], endometrial stem cells (EnSCs) [26]. Moreover, muscle-derived stromal cells (MDSCs) [27] showed a good in vitro potential in providing osteoprogenitor cells for fracture healing. Another powerful source can be seen in chemically defined medium pre-treated human periosteum derived stem cells (hPDSCs) that showed a great osteochondrogenic potential and brought to functional cartilagineous and mineralized tissue [28]. Finally, in addition to multipotent stem cells source, some studies tested the induced-pluripotent stem cells (iPS) differentiation to osteoblast-like cells. Nevertheless, the several limits in the iPS differentiation protocol and the related-risk of teratoma formation, do not allow their usage in clinical practice [29]. All these cells and their features are summarized in Table 2.

### 2.2. Stimuli

To increase the osteoconductive activity, during the years, scaffolds were often bioactivated by the administration of chemical conditioning such as molecules. Indeed, usually the cells are seeded in the scaffold enriched with growth factors, including fibroblast growth factor, bone morphogenetic proteins (BMPs), vascular endothelial growth factors, insulin-like growth factors, parathyroid hormones, Hedgehog, Wnts/beta-catenins, platelet-derived growth factors, prostaglandins, and the transforming growth factor β (TGFβ) [30,31,32]. The most used are the BMPs, that are known to favourite osteoblast differentiation. TNF-α and IL-6 factors are also able to stimulate the synthesis of bone tissue starting from MDSCs with the particular attention to the dose dependent response of MDSCs to TNF-α. Although the chemical stimuli increase the therapy efficacy, they involve some limits, such as tumours formation, that make the physical stimuli the most promising strategy to be applied on patients. In this context, research has been focused on developing scaffolds able to mimic the native structure, and consequently the function of the physiological tissue.

### 2.3. Scaffolds as Cell Support

In the bone tissue engineering, the scaffolds have to mimic the extracellular matrix physical properties in order to facilitate the cells recruitment, adhesion, proliferation and differentiation [33,34]. As summarized in Table 3, a great variety of scaffold has been developed in accordance with the main requirements of cytocompatibility, osteoinductivity, osteoconductivity. Moreover, in order to obtain an effective tissue regeneration, an optimal scaffold should guarantee dimensional stability, bioactivity, biodegradability and it needs to be easily manufactured and processed [35,36]. Nowadays, it is well known that cells activity and differentiation are affected by extracellular matrix stimuli [37]. In this context, the necessity of a matrix able to replicate both the structure and the mechanical proprieties of native bone emerges, mainly in terms of compressive strength and modulus [38]. Additionally, to guarantee mechanical stability and access for nutrients–metabolites diffusion, the scaffold structure needs to fit specific requirements related to the porosity [39,40]. In particular, the minimum pore size to support cell ingrowth is considered to be ~100 μm. However, pore sizes bigger than 300 μm are recommended to enhance vascularization and, therefore, osteogenesis. Indeed, small pores favored hypoxic conditions supporting the osteochondral formation before osteogenesis [41]. Moreover, high porosity (>90%) is required to guarantee both adequate nutrients diffusion and sufficient surface area for cell–biomaterial interaction [42]. This is the reason why, nowadays, in the field of bone tissue engineering, a huge effort has been spent in searching an optimal compromise between the high required mechanical strength and the inversely proportional porosity.

In accordance with the necessary features of osteoconduction and osteoinduction, several bone graft options such as autografts, allografts and bone graft substitutes are already in use in clinical procedures [43]. Nevertheless, even if currently considered as the gold standard, autograft application is limited due to donor site morbidity, supply scarcity, immunogenicity, risk of infection and injuries during harvesting. Moreover, the autograft has limited ability to accelerate normal healing and remodeling. If allograft overcomes the harvesting site-related drawbacks, in any case it still presents low osetoinductivity, lack in the osteogenic properties, great incidence of fracture and risk of infection, disease transmission or rejection [36]. In order to circumvent these limits, a great effort has been focused on the study of synthetic bone graft substitutes. Among the several tested biomaterials, polymers constitute a promising material mainly due to their cytocompatibility and biodegradability. In addition, polymers are characterized by flexible design, structures and chemical composition, which allow them to span a wide range of properties able to fit custom requirements [39]. In light of that, it is important to report that several polymers materials, mainly collagen- or PLGA-based materials, have already reached the clinical application (i.e., BioMed^®^ or Calcitek, Mucograft, Matriderm, GC membrane, etc.) [44]. Despite the huge effort in studying new technologies and materials able to fit specific size and shape, the surgical invasiveness of the 3D rigid scaffold remains a great issue. In this context, the hydrogel scaffolds, ideally injectable, represent a new promising strategy for minimal surgical implantation. Thanks to their high flexibility, the hydrogels can establish tight contacts with the host tissue, limiting fibrosis and favoring the osteoconductivity. In addition, due to their hydrophilic nature, hydrogels are able to absorb water up to 1000 times their original weight supporting cells growth, transplantation and proliferation and favoring the oxygen and nutrients permeability [45]. Moreover, their usage reduces the surgical operation time, minimizes the post-operative pain and scar size, achieves rapid recovery and reduces the clinical costs. In addition, the hydrogels can be filled with bioactive molecules and/or cells, that can directly take part as building blocks in tissue regeneration or just stimulate host response [46,47]. Despite several advantages, hydrogels are characterized by low mechanical properties that favours their application in lesion filling rather than in load-bearing lesions. In this review we are going to discuss the most so far studied polymers, spanning from natural to synthetic polymers (Table 4).

## 3. Natural Polymers

Natural polymers mainly involve proteins (collagen, silk fibroin) and polysaccharides (chitosan, alginate, hyaluronic acid, and cellulose). They usually contain bio-functional molecules that guarantee bioactivity, biomimetic surface and natural remodelling. On the other hand, their main drawbacks, such as immunogenic response, microbial contamination (i.e., endotoxin), reduced tunability, uncontrollable degradation rate, and weak mechanical strength, limit their application in bone tissue regeneration [44].

One of the most studied natural polymers for biomedical application is the collagen. As basic component of several animal tissues, it offers a number of favourable binding sites for bone cell adhesion, and it is known to promote the deposition of mineralized matrix. Its main features are the enzymatic biodegradability and the versatility in being processed in different physical forms, such as fibrous scaffold and hydrogel [39,44,48,49]. In 1997, Mizuno and colleagues reported collagen I ability in osteogenesis, starting from BMSCs cells [66]. Later, other in vitro studies confirmed the osteoblasts proliferation and osteogenic differentiation in radially oriented collagen scaffolds [67]. Considering the relevant collagen results in terms of osteoinductivity, several studies tried to overcome the collagen low mechanical properties. A possible strategy was the collagen use as a minor component in the scaffold. For instance, Calabrese’s research demonstrated the osteogenic activityof collagen, seeding human mesenchymal stem cells isolated from adipose tissue on collagen/Mg doped hydroxyapatite scaffold (70%). They verified bone augmentation and increased osteogenenis in in vivo mouse trials [68]. Indeed, even in absence of additional factors, the collagen insertion increases the scaffold ability to differentiate human adipose derived stem cells into mature osteoblasts. Another widespread strategy to improve the polymer mechanical properties requires the addition of inorganic compounds. For instance, a study tested in vitro cultures involving human foetal osteoblasts seeded on a nanofibrous hydroxyapatite/collagen/chitosan scaffold. They obtained relevant osteoblast proliferation, mineral deposition and alkaline phosphatase expression in comparison with the controls [69]. Following the ideal scaffold requirements, other researches changed the scaffold design obtaining a collagen-hydroxyapatite matrix characterized by optimal porosity for cell infiltration attachment and osteogenesis. Their usage in a mouse model treated with hydroxyapatite-collagen scaffold cellularized with mouse bone marrow derived MSC, showed excellent osteogenesis and cell infiltration in the 3D structure, with consequent total filling of the defect. In particular, from X-ray microtomography test, they reported 93% of porosity, 99% of interconnectivity and mean pore size about 101 μm. Indeed, the inclusion of hydroxyapatite not only increases the stiffness of the structure, but also facilitates pore interconnectivity and scaffold porosity, guaranteeing mineralization and healing of critical-sized bone defect, without any contraindication in the scaffold degradation rate [70,71]. Similar results were achieved substituting the hydroxyapatite with natural calcium phosphate nanoparticles [72]. Unfortunately, despite the improvements in mechanical properties that resulted suitable for the mouse model, the collagen-based scaffold mechanical strength was still not sufficient to the human load-bearing application. To further increase the mechanical properties, researches focused on the improvement of technological manufacturing strategies. In 2018, an innovative technique combined compression moulding hydroxyapatite reinforcements and paraffin microspheres, within a suspension of concentrated collagen fibrils. Researchers leach-out the microspheres and cross-linked the collagen matrix to increase the strength of the obtained material reaching a compressive modulus about 1 Mpa, almost one order of magnitude higher than the first generation one. Moreover, the structure showed interconnected pores of 300–400 μm of diameter and overall porosity of 85–90%. An in vitro test performed onto this new scaffold generation, cellularized with murine adipose-derive stromal cells, reports good cells infiltration and differentiation after 14 days from seeding. The angiogenesis and the osteogenesis abilities of this strategy have been also confirmed in mice model [73]. Both mechanical and biological properties favour the collagen-hydroxyapatite scaffold approaching to the clinical trials phase. Indeed, a prospective clinical trial involved thirty-four patients with knee chondral or osteochondral lesions of the patella, treated by cell-free scaffold implantation. Researcher and clinician evaluated osteochondral tissue regeneration with magnetic resonance imaging. The results demonstrated clinical improvement at short-term follow-up for the treatment of patellar cartilage defects [74].

In addition to collagen application as a 3D rigid scaffold, it has been involved in hydrogel studies. Due to the collagen hydrogel swelling ability in water and its high-water content, the collagen-based hydrogel facilitates mass transport and diffusion that make it a promising candidate for cell encapsulation. For example, this fact was demonstrated using adult human bone marrow derived stem cells, encapsulated in chitosan-collagen hydrogel. While the collagen presence guaranteed an appropriate cell spreading and proliferation, and conferred more compactness to the matrix, the chitosan increased alkaline phosphatase activity and calcium deposition in the osteogenic medium [75]. Considering these aspects, the collagen-chitosan composite hydrogel material resulted suitable either for cell encapsulation and delivery or for in situ gel forming application. More recently, collagen I-based hydrogel has been validated for drug-release usage as reported by Nabavi and colleagues. Indeed, their tests in rat model, using tacrolimus-loaded collagen hydrogel, reported appropriate scaffold porosity, swelling and drug release [76].

In order to obtain gelatins maintaining cytocompatible and biodegradable characteristics, along with properties that help in cell processes (i.e., migration to differentiation and proliferation), it was found another collagen application involving its denaturation from triple-helix into a single strand to produce the gelatin material [8,44,50]. As some collagen-hydroxyapatite scaffolds, porous gelatin-based scaffolds are obtained by freeze-drying technique. These scaffolds showed that changes in gelatin and crosslinking agent concentration guarantee pore size tunability, high level of porosity and different degradation rate [77]. Due to its thermosensitive characteristics, its main application is wound dressing and cells/molecules/drugs delivery. For instance, gelatin methacrylamide with embedded cartilage-derived matrix particles in subcutaneous rat model showed succesfull template remodelled into mineralized bone tissue [78]. In order to improve the gelatin-based scaffolds mechanical properties, gelatin was also tested in combination with other materials, suitable to increase the efficacy in bone repair application. For instance, gelatin methacryloyl/hydroxyapatite hydrogel (with and without embedding of cartilage-derived matrix particles) and gelatin/tricalcium phosphate showed relevant osteocunductive performance in in vivo rat trials that make them promising materials in bone tissue regeneration [79]. Moreover, other recent in vitro and in vivo evidences, presented the nanoscaled β-tricalcium phosphate/gelatin composite scaffold as a promising matrix for repairing the resected bone tissue in primary or metastatic bone sites [80,81]. Nevertheless, further studies are required to optimize the mechanical features in order to guarantee suitable support for the osteoblasts activity.

Another widespread used natural polymer is the silk fibroin. It is the structural protein of silk fiber, characterized by high cytocompatibility, low immunogenicity, limited bacterial adhesion and outstanding mechanical properties able to support osteogenic differentiation. Moreover, its biodegradability can be tuned varying molecular weight, crystallinity, and β-sheet structure [39,45,51,52]. The silk fibroin versatility results evident from its several applications that range from the silk as bulk component to the silk as coating of non-cytocompatible scaffold or as reinforcing elements. In the first case, for example, porous silk fibroin scaffold showed a hierarchical organization similar to the physiologic extracellular matrix characterized by high porosity and controlled pore sizes (200–400 nm) [82]. In vivo tests using silk fibroin membrane in rabbit calvarial model, reported a complete bony union across the defects after 8 weeks. To further improve the silk fibroin osteogenesis induction, other researchers covered the bulk structure with different materials. For instance, Wu et al. used BMP-functionalized-graphene oxide, obtaining increased osteogenic potential and bone formation in in vitro and in rats critical-sized calvarial bone defects, respectively [83]. Instead, other researchers tested nanohydroxyapatite-coated silk substrates in rabbit model. They reported good scaffold stability, cell attachments and new bone formation in four weeks [84]. The hydroxyapatite inclusion has been also used to enhance the mechanical properties of the silk fibroin scaffold, promoting mesenchymal stem cells differentiation and bone regeneration [85]. Still combined with hydroxyapatite, the silk fibroin has been also used as reinforcement in injectable bone cement. From both in vivo and in vitro studies, silk fibres inclusion improves the compressive strength and reduces the setting time with no negative effect on the injectability and cytocompatibility [86]. From a different point of view, other researchers focalized their studies on the silk usage as coating for composite scaffold [87,88]. Consistent with previous results, Kweon et al. tested hydroxyapatite-silk combination coating. Trials in rabbit model showed superior bone formation and tissue integration in this scaffold compared to the all the controls (i.e., uncoated scaffold, the hydroxypatyte-coated scaffold and the hydroxyapatite-collagen combination-coated scaffold) [88]. Instead, Li et al. designed a polycaprolacton nanofibers scaffold coated with silk and added to biphasic calcium phosphate. Thanks to the enhanced scaffold mechanical properties obtained by the multiple coatings, in vitro tests showed an increased proliferation and enhanced osteogenic differentiation of human mesenchymal stem cells. In particular, among the three scaffolds with increased number of coating (1,5,7), the 5X multiple coated scaffolds showed optimal combination of structural and mechanical properties for bone regeneration. Even if far from native bone mechanical stiffness, the 5X coating led to a relevant increase in compressive strength (0.3 MPa), still maintaining high level of pore interconnectivity and a porosity level (80%) consistent with the trabecular bone one [87]. In general, silk fibroin revealed a bone formation efficacy comparable with the commercial membranes that makes it one of the most promising material for medical application and in particular for bone regeneration [51].

Among the natural polymers, the chitosan represents another valid candidate usable in bone tissue regeneration. It is a linear positively charged polysaccharide consisting of randomly distributed N-acetyl glucosamine and D-glucosamine linked by (1,4) β glycosidic bond. Due to its charge, chitosan facilitates the interaction with several negatively charged molecules and membranes. Chitosan is characterized by cytocompatibility, biodegradability, non-toxicity, and mucoadhesivity [8,53]. Moreover, it promotes osteoblasts growth and matrix mineralization. Unfortunately, as much as collagen, the limit in mechanical strength requires its combination with different materials (e.g., calcium phosphate, hydroxyapatite, silk etc.). An explicative example is represented by a mixture of hydroxyapatite-chitosan and gelatin that returned a compressive strength (1.2 MPa) of these scaffolds close to the lower limit of compressive strength in spongy bone [89]. To further increase the osteoactivity, several studies tested the addition of the BMP chemical factor. For instance, in vivo studies showed the effect of chitosan-collagen scaffold with insertion of poly-L-lacticde-co-glucolide microsphere filled by BMP. The BMP release from the spheres improved the bone formation and the osseointegration in dog models, in four weeks [90]. Similar results have been reported in presence of another BMP-loaded scaffold that involved the chitosan material in form of microspheres embedded in absorbable collagen sponge. After 12 weeks, in rabbit model, the scaffold showed complete healing and recanalization of the bone-marrow cavity [91]. Moreover, the chitosan is also suitable in drug delivery field, as evident from both the in vitro and in vivo results. In particular, while adipose-derived stem cells, cultured on matrix of poly(L-lactic acid)/nanohydroxyapatite/alendronate-loaded chitosan microspheres, showed good drug release and enhanced osteogenic differentiation, in vivo results on rabbit models confirmed the osteogenic effect showing total bone repair within eight weeks [92]. All these results confirmed the chitosan suitability for drug delivery application, suggesting its possible application in hydrogel form. In particular, the recent improvements in manufacturing techniques led the researches in testing the chitosan in the form of bioprinted hydrogel structure. The chitosan-hydroxyapatite hydrogel was mixed with mouse calvaria-derived pre-osteoblasts (MC3T3-E1) cell and used as bio-ink for 3D printing. Mechanical analysis showed that these new scaffolds maintain viscoelastic properties and stability in physiological condition. The researches also verified the cell viability and the expression levels of osteogenic markers, confirming the successful mineralization and osteogenic cell differentiation in 21 days of culture [93].

In contrast to chitosan, alginate is a negatively charged polysaccharide. It is composed of (1,4)-linked β-D-mannurronic acid and α-L-guluronic acid whose changes in percentage provide tuneable mechanical and biological properties, making it a very interesting substrate [54,55]. This aspect, in addition to its cytocompatibility and its controlled gelation, makes the alginate widely use in minimally invasive bone-tissue engineering application, as well as in drug or cells delivery. As previously reported for chitosan, also the sodium alginate can be manufactured in microspheres form. For example, in drug delivery field, Bi and colleagues tested sodium alginate microspheres that, combined with chitosan and hydroxyapatite, have been loaded with doxorubicin hydrochloride drug. In vitro results validated the implied microspheres as a promising support for bone regeneration and controlled drug release system [94]. Other researchers used the alginate microspheres to encapsulate and protect the human umbilical cord mesenchymal stem cells. To improve the mechanical properties of the microbeads, they developed an injectable and mechanically strong stem cell construct combining the obtained microbeads with calcium phosphate cement. The mechanical properties of this construct matched with the trabecular bone properties (Young’s modulus is about 0.7 GPa) with positive effects on osteodifferentiation and bone minerals synthesis [95]. In light of the positive effects induced by the mechanical feature improvement, some researchers tested alginate-hydroxyapatite scaffold in both in vitro (with rat osteoblastic cell line) and in vivo (using rat models) conditions. Results confirmed an increment in cell adhesion induced by hydroxyapatite insertion and correct new bone formation. In particular, Lin et al. tested different alginate/hydroxyapatite ratio aiming at obtaining bone-like structure. The rat osteosarcoma cells displayed better cell attachment and proliferation in both 75/25 and 50/50 alginate-hydroxyaptite ratio, than the pure alginate one. Although both the obtained scaffold showed well interconnected porous structure with average pore size of 150 μm and over 82% porosity, the 50/50 alginate/hydroxyapatite scaffold prepared at −40 °C reported the best mechanical strength (compressive modulus about 18 MPa and the strength one around 150 MPa) that make it the most promising for bone application [96]. Consistently, in vivo tests performed with hydroxyapatite-alginate biocomposite in rats model showed high performance in terms of bone formation [97]. From opposite point of view, the alginate has been utilized to increase cell activity in injectable cement. After three months of cellularized scaffold implantation in rabbit, results showed that the alginate-chitosan loaded cement induces better bone formation in comparison to the simple cement implant [98]. Still, in coherence with the already presented polymers, the alginate has been tested in combination with BMP chemical factor in order to stimulate the cell activities. In vivo results with BMP-loaded-alginate-chitosan nanocomposite scaffold showed rat calvarial defect total closure after 16 weeks [99]. Finally, the increasing necessity of mini invasive surgical procedures required a huge effort in injectable material developing. In this context, injectable alginate has also been recently tested in combination with the fluorenylmethoxycarbonyl-diphenylalanine peptides. The rigid, still injectable, hydrogel showed excellent mechanical properties and a nanofibrous architecture similar to the natural fibrillar bone one, guaranteeing excellent in vitro results in terms of cell viability, osteogenic differentiation and cell adhesion to the hydrogel fibres, using MC3T3-E1 cells [100]. Moreover, the alginate hydrogels has been involved as bioink for bioprinting in bone tissue regeneration field [101]. The excellent alginate properties were confirmed by a comparative study of hydrogels in bone tissue engineering that reported the alginate superiority in comparison to hyaluronic acid in bone tissue engineering, reveled by cell viability, proliferation, calcium content, osteocalcin level and osteogenic differentiation [102].

Hyaluronic acid is a linear anionic glycosaminoglycan characterized by cytocompatibility, enzymatic biodegradability and viscoelasticity. Its major application is as hydrogels, even if sponges and cryogels forms represent a valid alternative for hyaluronic acid-based tissue engineering application [56]. Hyaluronic acid is known to regulate the cell differentiation and bone formation as confirmed by in vitro study showing N-cadherin modified hyaluronic acid ability in osteodifferentiation of human mesenchymal stem cells [57]. The already mentioned advantages induced by inorganic material inclusion led the researches in testing different composed materials. For instance, hyaluronic acid-gelatin hydrogel loaded into a biphasic calcium phosphate scaffold, showed highly interconnected porosity, with an average compressive strength (around 2.8 MPa) acceptable for trabecular bone substitution [103]. Consistent with the good mechanical properties, in vitro studies using bone marrow mesenchymal stem cells, exhibit high cell proliferation and alkaline phosphatase activities. Moreover, from in vivo studies on New Zealand white rabbits, the scaffold degradation resulted compatible with bone regeneration, beginning within three months after implantation and a high rate of collagen mineralization was verified [103]. Another application for hyaluronic acid is as molecular carrier. For instance, in 2008 a composed biomimetic bioglass-collagen-hyaluronic acid phosphatidylserine scaffold was developed. In vitro tests seeding human mesenchymal stem cells on these substrates showed higher degree of cell adhesion, proliferation and migration capability than those on the controls (a bioglass-collagen-hyaluronic acid scaffold) as demonstrated by gene expression of alkaline phosphatase, osteocalcin and osteopontin [104]. Other interesting studies performed both in vivo and in vitro tests on the poly-l-lysine/hyaluronic acid hydrogel enriched by curcumin and BMP-2. Human osteosarcoma derived cells exhibited good viability, proliferation, ALP activity and calcium deposition. In comparison with other polymers, the high bone formation induced by low burst followed by sustained release, guaranteed good results in in vivo tests, too [105,106]. In another study, the hyaluronic acid has been combined with chondroitin 6 sulphate and dermatan sulphate. After 21 days from hydrogel injection in parietal bone of male Wistar rats, almost complete bone healing occurred. Moreover, the neovascularization and the well-organized trabecular bone confirmed this mixture as promising material for bone tissue regeneration [107]. Another example is a trial involving culture of human adipose derived mesenchymal stem cells encapsulated and cultured in 3D using heparin-hyaluronic hydrogel. The cellular activity of encapsulated cells in 3D culture exhibited efficient cell spreading, proliferation, migration, and differentiation in comparison with those in the control scaffolds (heparin-PEG hydrogel and PEG-hyaluronic acid). This result corresponds to an effective degradation of the scaffold, necessary for 3D cellular activity. Therefore, the combination of heparin, as a binding domain for the cells, and hyaluronic acid, as a degradation site for cell secreted enzymes, constitutes an efficient scaffold for drug-release application and cell culture [108]. Other researchers confirmed the interesting characteristics of this material developing a new scaffold-type made of graphene oxide–chitosan–hyaluronic acid and enriched by simvastatin drug to favourite osteoblast differentiation. In-vitro analysis reported increased osteogenesis (osteoblast adhesion and proliferation) and scaffold mineralization in drug loaded scaffold [109].

From this state-of-the-art analys it results evident the main hyaluric acid application as molecules/drug carrier, favorited by its attitude in hydrogels forming [58].

To conclude the natural polymer section, cellulose represents another interesting material mainly obtained from different natural source such as bacteria, tunicates, and plants. Constituted of a polysaccharide macromolecule with β-(1,4) glycosidic bonds, it is characterized by hydrophilicity, cytocompatibility, bioactivity and optical transparency that make it suitable in several medical applications, such as skin tissue repair, cortical implants, drug delivery, vascular graft and medical implants [59,60]. In particular, in the bone tissue field, it is widely used due to its tunability in terms of chemical, physical, and mechanical properties [38,59]. Cellulose applications range from membrane, scaffold bulk material, coatings, nanofibers, films, and nanocrystals that lead the research in developing new technologies strategies [59,61,110]. The great cellulose bioactivity was verified in in vivo tests, where the bacterial cellulose membrane (0.10 mm) involved in the bone regeneration guiding, returned higher bone formation compared to the collagen one [111]. Other evidence has been reported by bone regeneration in rats femoral bone, where the cellulose was involved as bulk material [112]. In the same context, other researches tried to optimize the scaffold in terms of structure architecture by an innovative technique. In particular, a natural cellulose-chitin nanofibrils based scaffold was obtained by reverse templating of hydrogel scaffolds. The procedure starts with the sacrificial template preparation by layered micromirror lithography, its filling with cellulose and chitin nanofibrillar hydrogels, and finally its dissolution. The micromirror technique returned an extremely precise structure characterized by mathematically defined pore geometries able to mimic the natural bone structure. The suitability of the 3D structure was verified by human mesenchymal stem cells, that showed good adhesion and osteogenic differentiation [113]. Another successful technique implemented for the cellulose scaffold porosity optimization, is laser ablation on cellulose acetate electrospun fibres. The obtained scaffolds were characterized by pores with diameters ranging from 50 and 300 μm, that guaranted good osteoblast cell adhesion in in vitro tests. In addition, the same scaffold was mineralized by microablation (using phosphate buffered saline), resulting in crystals with a calcium/phosphate ratio around 1.56. These data are comparable with the hydroxyapatite one. The combination of laser ablation and mineralization showed an increment of osteoblast adhesion at the edges of the pores and overall enhanced mineralization [114]. Moreover, as the silk fibroin material, the cellulose has been also used as reinforcement. For instance, in vitro tests reported the cellulose nanocrystals insertion in maleic anhydride grafted poly(lactic acid) scaffold. Obtained results showed that the cellulose inclusion increased features compared to the two controls (i.e., maleic anhydride grafted poly(lactic acid) and cellulose nanocrystal-PLA)) demonstrated by both the relevant tensile strength (>10 MPa), and the improved stability during in vitro degradation. In particular, in a month, the lost mass of maleic anhydride grafted poly(lactic acid) scaffold reached 26.5 wt %, while the cellulose nanocrystal-PLA scaffold lost 40 wt %, and the maleic anhydride grafted poly(lactic acid)/ cellulose nanocrystal lost only 19.9 wt % [115]. In the same context, another in vitro study reported PLA reinforced by Poly(ethylene glycol)-grafted cellulose nanocrystals. Even if the addition of cellulose nanoscrystals didn’t achieve an improvement in tensile stress due to both their poor dispersion and the poor interfacial adhesion with PLA, it guaranteed improved strength of composite fiber mats. Optimal results have been achieved in 5% cellulose nanocrystals loading condition that showed improved cell viability and proliferation cell count [116]. The cellulose reinforce attitude was also tested in cellulose nanofibrils enriched gelatin scaffold by Gorgieva and colleagues. They obtained suitable structure characterized by both increased compressive strength (from 0.025 to 0.4 MPa) and elastic modulus (from 0.04 to 0.15 MPa) that, compatible with the osteoid one, favoured the mesenchymal stem cells growth and extracellular calcium deposition. In addition, the introduction of 3-aminolpropylphosphoric acid moieties into the same scaffold returned enhanced deposition of hydroxyapatite-like crystals, index of better mineralization [117]. From an opposite point of view, in order to increase the cellulose mechanical properties and bioactivity, other researches followed the already discussed strategy combining cellulose with hydroxyapatite. Indeed, PCL-nanocellulose fibrous matrix showed increased hydrophilicity after biomineralization by immersion in simulated body fluid [118]. Instead, from mechanical point of view, Eftekhari and colleagues proposed a nonocomposite material made by natural cellulose microcrystals, hydroxyapatite nanoparticles and poly l-lactide acid for bone regeneration applications. They obtained structures and mechanical properties (such as compressive yield stress of 2.2 MPa and Young’s modulus of 38 MPa) compatible with the lower limit of trabecular bone [119]. Still combined with hydroxyapatite, in order to obtain hydrogels, other researchers tested hydroxyapatite nanoparticles-absorbed nanofibrous 2,2,6,6-tetramethylpiperidine-1-oxyl-oxidized bacterial cellulose (HA-TOBC), with the addition of gelatine. The increment of the Young’s modulus, induced by both gelatin and hydroxyapatite, improved the calvarial osteoblasts proliferation and differentiation confirming the composite material as a potential candidate for the use in bone tissue engineering [120]. Consistently, some other in vitro and in vivo tests, performed on gelatin-modified bacterial cellulose scaffolds coated with hydroxyapatite, showed good biocompatibility and osteoinductivity [121]. Similar hydrogels took advantage from cellulose degradation for drug delivery application. In particular, the Gelatin-nanofibrillar cellulose-β tricalcium phosphate hydrogel scaffold, loaded with 0.5 μM Simvastatin (statins drug), showed gradually and controlled drug release, osteoblastic differentiation, new bone formation and collagen matrix deposition confirmed by both in vitro and in vivo tests. Therefore, the cellulose contribution in favoring the controlled drug release by slower degradation rate and the Gelatin-β tricalcium phosphate osteoconductivity, guarantee enhanced osteogenesis that makes this scaffold suitable for the bone tissue engineering [122]. Lastly, a peculiar cellulose application takes advantage from its attractive physicochemical properties and cytocompatibility, in order to favourite tissue integration to not-bioactive surface. For instance, fibrous cellulose nanocrystals interconnected with bioactive glass are used to cover stainless steel. In vitro studies confirmed cell activity acceleration in terms of attachment, differentiation and mineralization [123]. On the other hand, the same cellulose surface has been enriched with BMP-2-coating to further increase the cell activity. In vivo tests reported more bone formation and higher calcium concentration than the scaffolds alone [124].

In conclusion, the excellent cellulose capacity in cell adhesion, growth, osteoblast differentiation and ossification promotion, in addition to its excellent mechanical properties, confirmed it as one of the most attractive material for tissue engineering applications and in particular for bone regeneration [125].

## 4. Synthetic Polymers

Synthetic polymers present tailored structure and properties by appropriate designing the polymers functional groups. These advantages guarantee predictable, reproducible and tuneable properties that can be varied according to the specific applications. For instance, their degradation rate could be altered acting on the chemical composition, the crystallinity and on the molecular weight. On the other hand, if compared to the natural polymers, they present reduced bioactivity, presence of cells recognition sites and osteoconductivity. In this context, several coatings such as bioceramic particles have been tested to improve their surface performances for bone tissue regeneration. Among the synthetic polymers, the most used are the aliphatic polyesters: Poly(ε-caprolactone) (PCL), polylactide (PDLA, PLLA), Poly(lactide-co-glycolide) (PLGA) [44].

PCL is a semi-crystalline, biodegradable, non-toxic aliphatic polyester, potentially involved in load-bearing applications due to its slow degradation rate (three or four years). Its main limit is the hydrophobicity, which causes problems in cell adhesion and infiltration [39,44,62]. In this context, several co-polymerization strategies have been tested in order to improve the bioactivity. For instance, in vitro test exhibited promising results for the 3D printed PCL/alginate composite scaffold. The pre-osteoblasts, seeded on these scaffolds, returned increased biological activities as osteogenic differentiation, cell viability, calcium deposition and cell-seeding efficiency in comparison to pure PCL scaffolds. Moreover, the addition of alginate to the PCL scaffold guaranteed higher hydrophilicity, with consequent increment in water absorption evaluated at 14 days [126]. Another group tested the combination of PCL with PLA in both in vitro and in vivo conditions. From architectural point of view, they obtained a structure with 96% porosity, where the pores interconnectivity allows the cells to migrate in the whole sample. Consistently, in vitro tests involving human mesenchymal stem cells showed homogenous scaffold cellularization and increased cell viability, osteogenic gene expression and apatite-like deposition, if compared with the pure PCL scaffold [127]. As already discussed for natural polymers, the most widespread strategy to increase the scaffold bioactivity involves the hydroxyapatite insertion. Coherently, in vitro tests using human osteosarcoma cells and mouse calvaria-derived pre-osteoblasts on PCL/hydroxyapatite fibrous matrices showed better cell activity (attachment, proliferation, differentiation) and mineralization than on PCL alone. In addition, in vitro results obtained on the same scaffold by the Chuenjitkuntaworn’s research group, showed that primary human bone cells increased mRNA expression of collagen type I and osteocalcin, and a higher amount of extracellular calcium deposition in comparison with the PCL control alone. Moreover, in vivo tests reported increased new bone formation in a mouse calvarial defect model [128]. More recently, the same researchers tested PCL/hydroxyapatite scaffold also seeded with three different stem cells types (bone marrow-derived mesenchymal stem cells, dental pulp stem cells, and adipose-derived mesenchymal stem cells). This study investigated the scaffold in vitro ability in terms of cell growth, gene expression, and osteogenic differentiation. The results suggested the PCL/hydroxyapatite substrates as good candidate for all the tested stem cells, but in particular for dental pulp stem cells that returned the highest level of extracellular calcium deposition [129]. To improve the cells activitiy in terms of bioactivity, the already excellent performances of the PCL/hydroxyapatite scaffold were further increased by adding the poly-dopamine [130]. Indeed, in vitro tests using human mesenchymal stem cells showed better results in terms of cellular attachment, angiogenesis and osteogenesis in comparison with the standard-PCL scaffold. It was also demonstrated that the scaffold properties tune with the poly-dopamine concentration, in fact, cell activity increases consistently with drug concentration increment [131]. Another valid strategy proposed the PCL scaffold inclusion of silicon-doped hydroxyapatite to increase the substrate bioactivity, and of carbon nanotubes to enhance cell adhesion and differentiation. The compressive strength obtained with this new formulation (4 MPa) is compatible with the trabecular bone and it reflects on good bioactivity, cell adhesion and spreading. In conclusion, these results make this scaffold interesting for further investigations [132].

Another synthetic polymer involved in bone tissue regeneration field is the PLA. It is characterized by several features fundamental for bone regeneration, such as cytocompatibility, thermal stability, and no-toxic degradation products [63]. Moreover, in this material, the thermal stability and the degradation properties can be tuned varying the choice and the distribution of stereoisomers within the polymers chains (L/D ratios) as well as to the molecular weights [133]. As the other polymers, PLA has been tested in combination with hydroxyapatite aiming at improving the mechanical properties. For instance, a PLA/hydroxyapatite scaffold obtained by 3D bioprinting technologies, reported microvascular-mimicking channel and good elastic behaviours. In vitro human mesenchymal cells trials demonstrated higher cells adhesion, proliferation and osteo-differentiation if compared to either the same scaffolds without the addition of hydroxyapatite or the scaffolds with larger micro-channels [134]. Over the years, other composed PLA-based materials also showed good results in bone tissue regeneration field. For instance, a group developed a nano-fibrous mesh composed of PLLA and gelatin. They seeded mesenchymal stem cells on the scaffold and observed an effective osteogenic differentiation. Considering the excellent in vitro results, the trial of this polymer reached the in vivo phase. For instance, Ren and colleagues showed that the nano-fibrous meshes increase the new calcified bone formation into rat cranial defects [135]. Other researchers, instead, tested PLA in combination with PCL. Hierarchical macro-porous biocompatible scaffolds composed by PLLA and PCL are stabilized by hydrophobically modified silica nanoparticles. The obtained structure was similar to the natural extracellular matrix. These scaffolds, cellularized with the mouse bone mesenchymal stem cells, provided good biocompatibility and cell adhesion. Moreover, in vitro tests suggest this kind of scaffold as a good drug carrier. In fact, the same scaffold loaded with 1.2 wt % of enrofloxacin provided both a rapid and complete drug release profile after 10 h [136].

Finally, the most widespread synthetic polymer material is the PLGA, a linear copolymer that combines poly-l-lactic acid (PLLA) and glycolic acid (PGA). In bone tissue regeneration field, the PLGA is preferred due to its degradation rate tunability, ranging from weeks to months, just varying the two monomers ratio. Nevertheless, its suboptimal mechanical properties, due to its amorphous structure, and its poor osteoconductivity, limit the cell adhesion to the scaffold and its usage in load-bearing applications [64,65]. In this context, PLGA requests the addition of ceramic or active glasses, most of all hydroxyapatite. Among the several techniques tested to produce 3D PLGA-nano-hydroxyapatite porous scaffolds, the particulate leaching with bio-ceramic particles was the most common one, providing an effective porosity control, by varying the size and the amount of the porogen. Unfortunately, this technique presents several disadvantages such as incomplete solvent elimination and lack of interconnectivity and open pore structure [64]. To overcome these issues, Kim et al. proposed a novel method for a polymeric/nano-hydroxyapatite composite scaffolds fabrication by gas forming and particulate leaching, in absence of organic solvents. The obtained scaffolds showed highly porous structures (average porosity 91%) and improved mechanical properties (compressive modulus 4.5 MPa, tensile modulus 27 MPa). Both in vitro (using osteoblast) and in vivo (using osteoblast-scaffold construct implanted into a mice model) studies showed higher osteogenic potential, cell growth and mineralization activity in the new generated scaffold [137]. A decade ago, in order to avoid the process high temperature, and therefore the PLGA degradation, particulate leaching was combined with melt-molding technique. A solution composed of PLGA, sodium chloride particles and different amount of hydroxyapatite was prepared by the conventional solvent casting, and then compressively moulded in a specially designed mould. The obtained scaffold showed high porosity (around 90%) and porous interconnectivity. The structure was characterized by both macropores (100–300 μm) created by the leaching step, and micropores (1–50 μm) in the pore walls, that increase consistently with the content of hydroxyapatite. The 20 wt % hydroxyapatite/PLGA sample exhibited the maximum mechanical features (compressive strength of 2.31 MPa) and high osteoblasts viability. The same scaffold in in vivo tests showed rapid healing in the defects that make it an optimal composite for bone repairing [138]. Still trying to increase the PLGA mechanical properties, in these years the research has made many other steps forward. For instance, the thermally induced phase separation was used for PLGA and nano-biphasic component (hydroxyapatite β-tricalcium phosphate powders), achieving the optimum value of Young’s modulus (15MPa in 50% PLGA nano-biphasic component ratio) [139]. Some other researchers instead, to meliorate the scaffolds architecture, proposed PLGA/nano-hydroxyapatite substrates developed via selective laser sintering. They obtained well-controlled pore architectures and high exposure of the bioactive ceramics to the scaffold surface [140]. To better investigate cell differentiation and gene-expression pattern during osteogenic differentiation, several cells (i.e., adipose-derived stem cells, mesenchymal stem cells and pulp cells) have been seeded on these nanofibrous scaffolds. The differentiated adipose-derived stem cells, showing the best results in terms of osteogenesis, suggested their suitable applicability in bone tissue regeneration [141]. Further, in vitro investigation with MC3T3 cells cultured on the nano-hydroxyapatyte/PLGA composite nanofiber scaffolds reported higher cellular adhesion, proliferation and enhanced osteogenesis in comparison to spherical-hydroxyapatite/PLGA and PLGA nanofiber scaffolds [142]. Instead of the fiber structure, another study showed in vivo tests using hydroxyapatite-coated PLGA in form of microspheres, seeded with rat osteoblasts and injected into a subcutaneous dorsum of the mice. The results indicated an increment in the new bone formation in presence of apatite-coated microspheres compared to pure PLGA ones [143]. The obtained results suggested the PLGA-hydroxyapatite microspheres in drug delivery context. In particular, the PLGA-hydroxyapatite microspheres were tested with alendronate, an osteoporosis-preventing drug: the controlled release increased the osteoblast proliferation [144]. Therefore, the results validated the PLGA/Hydroxyapatite-alendronate ability in the improvement of both osteoblast proliferation, and osteoinduction activity [64]. From another point of view, the PLGA was also testes as minor component (around 10%) in 3D printed hydroxyapatite-based scaffold (90% in weight). Even if lower than the pure hydroxyapatite scaffold, the composed scaffold reported good elastic properties (Young’s modulus of 11 MPa) and absorbent capacity, suitable for bone cells activities. In fact, in in vitro studies with bone marrow–derived human mesenchymal stem cells, the scaffolds showed good cell viability and proliferation, and induced osteogenic differentiation after four weeks. In addition, the researchers also verified the scaffold biocompatibility in mouse subcutaneous implant model, demonstrating the efficacy of these scaffold in bone formation both in rat posterolateral spinal fusion model and in a large non-human-primate calvarial defects case study. Similar results were obtained substituting the PLGA with PLA in scaffold preparation. Also in this case, they obtained promising results in terms of negative immune response, vascularization, tissue integration, osteinduction and new bone growth, confirming the PLGA potentiality in bone tissue regeneration despite its non-optimal mechanical properties [145]. Finally, in order to increase cell affinity and to generate a biomimetic interface some studies focused on the PLGA scaffold surface modification. For instance, today, it is well known that oxygen plasma treatment allows the formation of bone-like apatite thanks to negative charge and surface roughness. These aspects guaranteed enhanced adhesion cells and proliferation of osteoblast-like cells if compared to pure PLGA [146]. In order to guarantee cells adhesion, another group, investigated the hybrid nanofiber sheets prepared by PLGA and covalently linked to the arginine-glycine-aspartic acid, the minimal integrin-recognizable sequence. They obtained three-dimensional architecture-like natural ECM. In vitro tests using several cell sources (such as murine macrophage cells, murine pre-osteoblastic cells, human osteosarcoma cells, and primary human aortic smooth muscles cells) showed higher initial adhesion and increased proliferation compared to the pure PLGA [147]. Differently, Lee et al. chose another strategy inducing the surface bioactivity by immobilizing bone-forming peptide 1 derived from the BMP-7. Researchers seeded human mesenchymal stem cells culture on these new substrates and, after 14 days, implanted the cellularized scaffold onto mouse calvarial defects. Consistently, they reported a significant increment in bone growth and effective scaffold integration with the original tissue [148].

To conclude, even if the safety and tuneable degradation properties make the PLGA a suitable material for bone tissue engineering, the required mechanical properties and cell affinity, force its combination with hydroxyapatite and biomimetic surface coating.

In Table 5, we summarized the main achieved results using natural or synthetic polymers in combination with the different strategies adopted to increase the scaffold suitability in bone tissue regeneration.

## 5. Discussion and Conclusions

The elderly population increase, with a consequent increment in bone-related diseases, represents one of the major public health problems. Nowadays, in the most severe case, the conventional therapies involve metallic implants, autograft or allograft with many drawbacks related to fibrous tissue encapsulation, resorption and immune rejection, respectively. In this social context, the bone tissue engineering represents one of the most promising strategy to overcome the health problems, and nowadays the research has been focusing on scaffold optimization. The ideal scaffold usable in tissue bone regeneration should be characterized by optimal porosity, biodegradability, and specific mechanical and chemical properties able to guarantee at least extra cellular matrix-like support to the cells. In recent decades, polymers biomaterials, despite their low mechanical properties, received a great attention in this field, mainly due to their cytocompatibility, their tuneable properties and their processability. To circumvent their mechanical limits, remarkable advance has been made mainly mixing polymers with inorganic materials, such as hydroxyapatite or calcium phosphate (second column in Table 5). Indeed, in the last twenty years, both in vitro and in vivo studies showed optimization in terms of 3D structure (porosity, pore interconnectivity) and mechanical features that, being compatible with the bones ones, positively act on the osteoinductivity and osteoconductivity processes. Still aiming at ideal scaffold, the other most widespread strategy (third column in Table 5) involved chemical factors inclusion (for instance BMP) and drugs, able to favorite osteogenic potential and osteointegration. In this context, the improvement in manufacturing technologies lead to overcome the structured scaffold, in favor to hydrogel application. Indeed, the hydrogel structure guarantees minimal surgical invasion, reduced costs and fast recovery for the patient. Moreover, from a biological point of view, the hydrogel structure guarantees optimal cell infiltration, proliferation, migration with consequent benefits in terms of osteoconductivity and bone tissue integration. For all these reasons hydrogels represent one of most promining strategy in bone tissue engineering, although their suboptimal mechanical features still request further improvement for load-bearing application.

To conclude, in bone tissue engineering the analysis of the state-of-the-art revealed an increasing effort in testing different polymers/materials mixture and innovative techniques aiming to modulate cell interaction via biomimetic substrates. In this context, even if the perfect scaffold for bone tissue regeneration remains yet to be developed, in vitro and in vivo results revealed some promising insights into polymers for further research in bone tissue regeneration.

## Figures and Tables

**Table 1:** The native bone tissue physical characteristics, relevant for bone tissue engineering.

Bone	Young’s Modulus [8]	Compressive Strength [3]	Porosity [3,4]
Cortical	15–20 GPa	100–230 MPa	5–30%
Trabecular	0.1–2 GPa	2–12 MPa	30–95%

**Table 2:** List of stem cell types for bone tissue regeneration and their features.

Acronym	Cell Type	Feature
BMSC	Bone marrow stem cell	Largely available from the body, they enhance osteoblasts differentiation. Invasive extraction procedure.
ADSC	Adipose-derived stem cells	Largely available, they recruit other cells from the bone. Lower osteogenic potential than BMSC.
UMSC	Umbilical cord mesenchymal stem cells	Largely available and non-invasive procedures. Ethical problems correlated to their usage.
DPSC	Dental pulp stem cells	Easy harvesting. Fast proliferation and possible differentiation in different types of cells. Lower ostogenic potential than BMSC.
PDLSC	Periodontal ligament stem cells	Reduction of proinflammarory cytokines. They induce both osteoblast commitment and vascularization. They need conditioned medium.
GMSC	Gingival mesenchymal stem cell	Reduction of proinflammarory cytokines. They need conditioned medium.
DFSC	Endometrial stem cell	High proliferation rate but weak osteogenic potential.
JBMSC	Jaw bone mesenchymal stem cells	Highly expandable. Good osteogenic potential.
AMSC	Amnion mesenchymal stem cells	Anti-inflammatory multipotent cells. Non-invasive harvest. Limited availability.
MDSC	Muscle-derived stromal cells	Good osteogenic potential. Contrasting results between human and animal cells.
PDSC	Periosteum-derived stem cells	Large availability, they produce functional tissue. Difficult extraction procedure.
iPS	Induced-pluripotent stem cells	Possible teratogenic cells in vivo.

**Table 3:** Scaffold requirements for effective bone tissue regeneration.

Requirement	Description
Cytocompatibility	The released products should be non-toxic and non-inflammatory.
Bioactivity	Scaffold should interact with the tissue according to osteoinductive and osteoconductive principles.
Biodegradability	An ideal scaffold should degrade in a controlled way by external-enzymatic/biological process.
Suitable porosity	Interconnected pores are necessary for cell diffusion and migration. The scaffold should present micro porosity to guarantee enough surface area for its interaction with the tissue. Macro porosity is required for cell migration and cell growth. On the other hand, the porosity should not affect the mechanical stability.
Mechanical features	Scaffold should reproduce elastic and fatigue strength of the bones tissue site.
Tunable properties	Scaffold should have customizable properties.
Easy manufacturing, processing and handling	Scaffold should be easy to be fabricated and sterilized. Easy clinical manipulation is required.

**Table 4:** Natural and synthetic polymeric materials suitable for bone tissue regeneration and their main characteristics.

Scaffold	Advantages	Disadvantages	Ref.
Natural polymers	Bioactivity Biomimetic surface Natural remodelling	Immunogenic response Microbial contamination Weak mechanical strength Lack of tunability Uncontrollable degradation rate	[39,44]
Collagen	Similar to ECM Cytocompatibility Enzymatic biodegradability Cytocompatibility and cell-binding properties Versatility in being processed in different physical forms Possible injectability FDA approved	Low mechanical strength Difficult disinfection Difficult handling	[48,49]
Gelatin	Cytocompatibility Biodegradability Porosity tunability Osteoconductivity	Poor mechanical properties Low stability in physiological conditions	[8,50]
Silk fibroin	Cytocompatibility Immunogenicity Flexible processability Limited biological adhesion High mechanical strength Thermal stability Easy chemical modification		[51,52]
Chitosan	Cytocompatibility Biodegradability Cell-binding, differentiation and migration properties Antibacterial properties Mucoadhesivity Easy properties tunability	Poor mechanical strength and stability Rapid in vivo degradation rate	[8,53]
Alginate	Cytocompatibility Cytocompatibility Tuneable properties Easy gelling	Difficult to sterilize Low cell adhesion	[54,55]
Hyaluronic acid	Cytocompatibility Biodegradability Enzymatic biodegradability Viscoelasticity Easy manipulation Easy chemical functionalization	Poor mechanical strength Very rapid degradation	[56,57,58]
Cellulose	Hydrophilicity Cytocompatibility Bioactivity Optical transparency Tuneable properties		[38,59,60,61]
Synthetic Aliphatic polymers	Tailored structure Predictable and reproducibility properties Water solubility Tuneable crystallinity Tuneable physical and mechanical properties FDA approved	Reduced bioactivity No cell recognition sites Low osteoconductivity Possible adverse tissue reaction for acid degradation product Lack of cellular adhesion	[39,44]
PCL	Cytocompatibility Biodegradability Slow degradation rate	Hydrophobicity Low bioactivity	[62]
PLA	Cytocompatibility Thermal stability Tuneable properties		[63]
PLGA	Wide range of degradation rate Tunability	Suboptimal mechanical properties Poor osteoconductivity	[64,65]

**Table 5:** In vitro and in vivo experiments are classified according to the material and the strategy adopted with the aims of enhancing the scaffolds performances. In the first row the main strategies used to make the polymers suitable for bone scaffolds are reported. In the second row, the fundamental common aim for each strategy. All the analyzed studies in this review have been here classified according to both the material and the strategy adopted. In addition to the achieved general aim, the relevant evidences specific for each combination of material and strategy have been resumed in the table. Almost half of the reported studies overcame the polymers main limit of low mechanical feature by addition of inorganic materials (second column). This strategy not only guanteed an increased mechanical strenght but also favored the cells activities, as specifically reported for each material-strategy combination. In the third column, the several studies involving chemical factors/drugs addition to enhance the polymers osteoactivies. In the fourth column are reported the references where polymers - mainly the natural ones - are used as minor component to enhance the bioactivity or as reinforcement (only in silk and cellulose cases) of other bulk materials. Just few studies can not be included according to the described criteria and are reported in the firth column. The residual number of the studies in the “other stretegies” column confirmed the reported strategies as the most applied ones to obtain the ideal scaffold, that aim to both overcome the polymers limits and enchance their characteristics. The hydrogel/injectable matierals are indicated with ^1^.

Strategy	Inorganic Material Addition	Chemical Factor/Drug Addition	As Minor Component	Other Strategies
Main Aim	To increase the mechanical properties	To enhance osteoactivities	To increase cytocompatibility of other materials	
Collagen	Facilitate pore interconnectivity good porosity, cell infiltration, cell differentiation, angiogenesis and osteogenesis [69,70,71,72,73,74]	Appropriate scaffold porosity, swelling, and drug release [75,76]^1^ [90,91,104]	Increased osteogenesis, and osteoblast differentiation [68]	[66,67][75]^1^
Gelatin	Relevant osteoconductive properties [80,81,89] [79,103,122] ^1^	New bone formation [78,80][122] ^1^		[77]
Silk fibroin	Good stem cells differentiation, cells attachment, and osteogenesis [84,85,88][86] ^1^	Increased osteogenic potential [83]	Increased compressive strenght of bone cement; increased prolifertion and osteogenic differantiation [87,88]	[82]
Chitosan	Good osteogenic cell differentiation [89,92,94][93] ^1^	Suitable drug release and enhanced osteogenic differentiation [90,91,92] [75,93]^1^ [94,99,109]	[98] ^1^	
Alginate	Osteodifferentiation, increased cell adhesion and osteogenesis [95] ^1^ [94,96,97]	Good results in cell viability, osteogenic differentiationand cell adhesion and controlled drugs release [94,95,99]	Increased osteogenesis in bone cement [98]^1^	[100,101,102]^1^
Cellulose	Increased osteoblast proliferation, differentiation, and osteoconductivity [118,119,121][120,122]^1^	Controlled drug release, osteoblastic differantiation, and new bone formation [122]^1^ [117,124]	Increased compressive strenght and in vitro stability; improved cell attachement, viability, proliferation, and calcium deposition [115,116,117,123]	[111,112,114] [113] ^1^
Hyaluronic acid	Good porosity, proliferation and mineralization [103,105] ^1^	High cell adhesion, proliferaration and migration, vibility, and calcium deposition [104][105,105,106,107,108,109] ^1^		[102]
PCL	Increased cell attachment, proliferation, differentiation, calcium deposition, and bone formation [128,129,130,131,132]	Increased cellular attachment, angiogenesys, and osteogenesis [130,131]		[126,127]
PLA	Good cell adhesion proliferation, and osteo-differentiation [134,136]	Rapid and complete drug release, good cell viability, proliferation, and osteogenic differentiation [136]		[133,135]
PLGA	Good porosity, osteogenic potential, and mineralization activity, higher cellular adhesion/proliferatio, and new bone formation [137,138,139,140,141,142,144,145][143] ^1^	Higher initial adhesion, increased proliferation, new bone formation, and suitable scaffold integration; controlled drug release. [144,147,148]	Good cell viability, proliferation, and osteogenic differentiation [145]	[146]

^1^ Hydrogel/ Injectable material.
